# Survivorship wellness: a multidisciplinary group program for cancer survivors

**DOI:** 10.1007/s00520-023-08117-3

**Published:** 2023-10-26

**Authors:** Chelsea J. Siwik, Kinnari Jhaveri, Jamie Alexis Cohen, Mikela Barulich, Alison Chang, Anna O. Levin, Neha G. Goyal, Michelle Melisko, Margaret A. Chesney, Dianne Shumay

**Affiliations:** 1 Osher Center for Integrative Health, University of California, San Francisco, CA USA; 2 Helen Diller Family Comprehensive Cancer Center, University of California, 1600 Divisadero Street, Fourth Floor, San Francisco, CA 94115 USA; 3 School of Medicine, University of California San Francisco, San Francisco, CA USA

**Keywords:** Psycho-Oncology, Health behavior, Survivorship, Multidisciplinary

## Abstract

**Purpose:**

National mandates require cancer centers provide comprehensive survivorship care. We created an 8-session, group intervention, the Survivorship Wellness Group Program (SWGP), that covered 8 topics: nutrition, physical activity, stress, sleep/fatigue, sexuality/body image, emotional wellbeing/fear of cancer recurrence, spirituality/meaning, and health promotion/goal setting. This study examined the acceptability and preliminary outcomes of SWGP.

**Methods:**

We evaluated SWGP using questionnaire data collected at program entry and 15-week follow-up. Questionnaires assessed acceptability and impact on anxiety, depression, quality of life, and perceived knowledge of topics. Enrollees who consented to participate in research and completed the baseline and 15-week follow-up were included in the analysis (*N* = 53). We assessed acceptability and preliminary outcomes using paired-samples t-tests. Due to the COVID-19 pandemic, SWGP transitioned to telehealth partway through data collection. Post-hoc analyses compared outcomes by intervention delivery.

**Results:**

Participants completed an average of 7.44/8 classes. Participants reported a mean response of 3.42/4 regarding overall program satisfaction and 90.6% reported being “very likely” to recommend SWGP. SWGP was associated with decreases in anxiety and depression; increases in physical, emotional, functional, and overall quality of life; and increases in knowledge of all health behavior domains. No outcomes differed significantly between delivery in person versus telehealth.

**Conclusions:**

SWGP offers an acceptable and replicable model for cancer centers to meet national survivorship care guidelines.

**Implication for cancer survivors:**

SWGP provides a comprehensive service for cancer survivors post-treatment, and was associated with better quality of life, fewer mental health symptoms, and increased knowledge in multiple domains of wellness.

## Background

Cancer care standards mandate addressing the global needs of survivors post-active treatment [1–7], especially as the number of patients living through and beyond cancer grows [8, 9]. There is need for cancer care providers and institutions to deliver *survivorship care*: comprehensive post-treatment support that promotes long-term health and wellbeing. However, cancer centers face challenges providing care that meets the complex physical, mental, and spiritual wellness needs of survivors [9, 10]. Lingering treatment effects include fatigue, pain, impaired sexual functioning, and sleep difficulties [9, 11], which may be complicated by fear of recurrence, perceived loss of support, and existential distress [10, 12–14]. Existing services for cancer survivors may be fragmented [15], offering programs on singular topics such as nutrition, physical activity, or sexual health [16, 17]. New care models need to deliver comprehensive services that address the diverse concerns of a growing population of survivors at the critical transition of care post-treatment. In response, the UCSF Psycho-Oncology team created the Survivorship Wellness Group Program (SWGP), which is an evidence-based group intervention that our group described previously in a published commentary [18, 19]. This paper describes SWGP and presents an initial assessment of its acceptability and preliminary outcomes.

## Methods

### Survivorship wellness group program

SWGP is a manualized program facilitated by a multidisciplinary team (nutritionists, exercise counselors, medical chaplains, health psychologists, and health coaches) to address patients’ physical, psychosocial, and spiritual needs post-treatment. SWGP delivers education and skills training designed to address common concerns of cancer survivors [18]. The program’s weekly, 90-min group sessions address 8 topics: nutrition, physical activity, stress, sleep and fatigue, sexuality and body image, emotional wellbeing and fear of cancer recurrence, spirituality and meaning, and health promotion and wellness goal setting. To foster group discussion, social modeling, and behavior change reinforcement, the target group size is between 8–15 individuals. Sessions begin and close with a group check-in facilitated by a clinical psychologist to explore health behavior change efforts. The topic expert for the session (e.g., nutritionist) presents educational content and a health psychologist leads an experiential mindfulness or relaxation exercise. As needed, patients are offered referrals to additional survivorship services in individualized care clinics. For additional details on program content, see the previously published commentary [18].

SWGP offers sessions continuously, and survivors can join at any part of the cycle to complete all eight topics. The program is currently offered in English, and those who are unable to participate for language or other reasons are offered care alternatives. For purposes of routine clinical care and quality improvement, all patients complete a baseline questionnaire, and are sent a follow-up questionnaire at 15 weeks post-enrollment. Sessions are billed to insurance using a health and behavioral group code [18], and for those without insurance, SWGP is provided at no cost. With the onset of the COVID-19 pandemic in March 2020, the program transitioned from in-person to telehealth [19]. By Spring of 2020, 220 patients had participated in SWGP, and there was interest in evaluating the program’s effects. We initiated an evaluation of the program and hypothesized patients would have high satisfaction and attendance rates and the program would be associated with improved quality of life, reduced anxiety and depressive symptoms, and increased knowledge of health behaviors*.*

### Patients

SWGP is an active clinical intervention offered at UCSF Psycho-Oncology. Adult patients with a history of cancer (of any type) are eligible for SWGP after completing active treatment. To evaluate SWGP, we obtained IRB approval to obtain retrospective and prospective consent from patients. When we received IRB approval, 220 patients had participated in SWGP. Of the 220, patients who (1) consented to participate in research and (2) completed both the baseline and 15-week follow-up questionnaire were included in the current analysis. We were unable to retrospectively reach some of the prior participants by phone to obtain consents, which coincided with the beginning of the COVID epidemic, because we did not have a number on file (or another way to contact them) or because they did not return our call. We obtained consent to participate in research from ninety-two patients. Of the 92 patients who consented, 39 patients had not completed the 15-week follow-up questionnaire, yielding a final sample size of 53 patients who had completed both the baseline and 15-week assessments. Of the 53 patients, 20 participated in SWGP in person prior to March 2020, 6 transitioned from in person to telehealth due to the pandemic, and 27 participated via telehealth. Patients were referred to SWGP by their oncologist (*n* = 14), nurse (*n* = 7), another provider (*n* = 17), friend (*n* = 2) or from a flyer (*n* = 6), website (*n* = 1) or ‘other’ (*n* = 5). This study was approved by the Institutional Review Board of University of California, San Francisco (UCSF) and registered with ClinicalTrials.Gov.

### Measures

#### Sociodemographic and clinical information

Sociodemographic characteristics were assessed in both baseline and 15-week questionnaires. Information regarding primary cancer diagnosis and stage were obtained from participants’ electronic medical records. Participants reported on quality of life, depression, anxiety, and perceived knowledge of program topics on both baseline and 15-week questionnaires. The 15-week questionnaire also assessed program satisfaction and perceived goal achievement.

#### Quality of life, depression, and anxiety

The 27-item Functional Assessment of Cancer Therapy—General (FACT-G [20]) was used to assess physical, social/family, emotional, and functional quality of life during the past week. The four-item Patient Reported Outcomes Measurement Information System (PROMIS) depression and anxiety scales [21] were used to assess symptoms of depression and anxiety during the past week.

#### Perceived knowledge, goal achievement, and program satisfaction

Perceived knowledge about the eight SWGP topics was measured on baseline and 15-week questionnaires using a 5-point self-rating scale (1 = not at all knowledgeable, 2 = somewhat knowledgeable, 3 = moderately knowledgeable, 4 = very knowledgeable, 5 = extremely knowledgeable). Perceived goal achievement was measured on the 15-week questionnaire using a 5-point rating scale (1 = I have not met any of my goals, 2 = I have met few of my goals, 3 = I have met some of my goals, 4 = I have met most of my goals, 5 = I have met all my goals). Overall program satisfaction was measured on the 15-week questionnaire (1 = not at all satisfied, 2 = somewhat satisfied, 3 = mostly satisfied, 4 = extremely satisfied), as was likelihood participants would recommend the program to other survivors (1 = not at all likely, 2 = somewhat likely, 3 = very likely).

### Statistics

Analyses were conducted in Statistical Package for the Social Sciences (IBM SPSS version 27.0). Data were tested for assumptions of parametric data, including tests for normality, significant outliers, linearity, and homoscedasticity [22]. Non-parametric tests were used to analyze data that did not meet parametric assumptions.

To assess program acceptability, means and percentages were calculated for patients’ reported satisfaction and likelihood to recommend the program to others. Paired-samples t-tests were conducted to test change in physical, social, emotional, functional, and overall quality of life, and symptoms of anxiety and depression from baseline to week 15. Wilcoxon signed-rank tests were conducted to test change in perceived knowledge of nutrition, exercise, stress, sleep, emotional wellness, spirituality, sexuality, and goal setting from baseline to week 15. To assess perceived goal achievement at week 15, means and percentages were calculated using participants’ self-report.

#### Post-hoc analysis

Non-parametric (i.e., Mann–Whitney U tests) and parametric tests (independent-samples t-tests and two-way ANOVAs) were conducted to examine if outcomes differed between intervention formats (i.e., in-person pre- pandemic vs. telehealth post- pandemic). The six participants who received SWGP via a hybrid format due to abrupt transition were excluded from these analyses.

## Results

Demographic and medical characteristics are summarized in Tables 1 and 2.

**Table 1:** Demographic characteristics of the study sample (*N* = 53)

Variable	Mean (*SD)*	Range
Age	55.5 (*12.5*)	23 – 76
	Frequency (*n*)	Percent (%)
Sex		
Male	6	11.3
Female	47	88.7
Race		
White	42	79.2
Asian	7	13.2
Black	1	1.9
Other	2	3.8
Unknown	1	1.9
Ethnicity		
Not Hispanic or Latino/a	44	83.0
Hispanic or Latino/a	3	5.7
Other	1	1.9
Unknown	4	7.5
*	1	1.9
Education		
Some college or technical school	6	11.3
College graduate	26	49.1
Some graduate school	3	5.7
Master’s degree	12	22.6
PhD/MD/JD or equivalent	4	7.5
Prefer not to answer	1	1.9
*	1	1.9
Work Status		
Working full time (≥ 35 h/week)	10	18.9
Working part time (< 35 h/week)	9	17.0
Full time, parenting or caregiving	4	7.5
Student	2	3.8
Retired	16	30.2
On leave or disability	8	15.1
Other	3	5.7
*	1	1.9
Marital Status		
Single	10	18.9
Committed relationship	6	11.3
Married	31	58.5
Divorced	4	7.5
Prefer not to answer	1	1.9
*	1	1.9
Annual Income		
< $25,000	6	11.3
$25,000 – 49,999	1	1.9
$50,000 – 74,999	5	9.4
$75,000 – 99,999	1	1.9
≥$100,000	29	54.7
Prefer not to answer	10	18.9
*	1	1.9

*Denotes missing data

**Table 2:** Medical characteristics of the study sample (*N* = 53)

Variable	Frequency (*n*)	Percent (%)
Primary cancer diagnosis		
Breast	26	49.1
Gastrointestinal	10	18.9
Hematologic	5	9.4
Head and Neck	4	7.6
Gynecological	5	9.5
Genitourinary	1	1.9
Thoracic	1	1.9
Adrenal	1	1.9
Stage of primary diagnosis		
0	2	3.8
I	13	24.5
II	15	28.3
III	14	26.4
IV	6	11.3
*	3	5.7
Time since treatment completion		
Less than 1 month	2	3.8
1–5 months	28	52.8
6–12 months	9	17.0
More than 1 year	12	23.5
*	2	3.8

*Denotes missing data

### Acceptability

On average, participants completed 7.44 (*SD* = 1.20) of 8 classes. Many completed all 8 classes (72%), and the majority (96.2%) completed four or more classes. Participants reported a mean response of 3.42 (*n* = 52) on a scale of 1 – 4 reflecting their overall satisfaction with the program. No participant reported ‘not at all satisfied.’ Greater than 90% of participants reported feeling *extremely* or *mostly* satisfied with the program. Participants reported a mean response of 2.91 on a scale of 1 – 3 regarding the likelihood of recommending the program to others. No participant reported they were not likely to recommend the program and 90.6% of patients reported they were very likely to recommend the program.

### Preliminary outcomes

SWGP was associated with a significant increase in physical, *t*(51) = 3.02, *p* < 0.01, emotional, *t*(51) = 4.64, *p* < 0.001, functional, *t*(51) = 3.29, *p* < 0.01, and overall, *t*(51) = 4.58, *p* < 0.001, quality of life. Differences in social quality of life did not emerge as significant (*p* = 0.052); however, among those who completed at least 50% of the program (≥ 4 classes), SWGP was associated with a significant increase in social quality of life, *t*(49) = 2.22, *p* < 0.05. SWGP was associated with a significant decrease in anxiety, *t*(48) = 2.36, *p* < 0.05, and depression, *t*(48) = 2.82, *p* < 0.01. SWGP was associated with a significant increase in perceived knowledge of all eight program domains: nutrition (*n* = 52, *z* = 3.71, *p* < 0.001), exercise (*n* = 52,* z* = 4.30, *p* < 0.001), stress (*n* = 51,* z* = 4.80, *p* < 0.001), sleep (*n* = 51,* z* = 5.50, *p* < 0.001), emotional wellness (*n* = 51,* z* = 5.77, *p* < 0.001), spiritual wellness (*n* = 51,* z* = 5.24, *p* < 0.001), sexual wellness (*n* = 52,* z* = 4.87, *p* < 0.001), and goal setting (*n* = 52,* z* = 5.49, *p* < 0.001). Participants reported a mean response of 3.11 on a scale of 1 – 5 regarding their perception of having attained their stated goals at week 15. Most reported they had met some of their goals (47.2%), with only one reporting that they had not.

### Post-hoc analysis

After excluding those who received SWGP via a hybrid format (a mix between in-person and via telehealth; *n* = 6), a two-way mixed ANOVA revealed a main effect of group on anxiety, such that there was a statistically significant difference in anxiety between patients who received the program in person (*M* = 6.58, *SD* = 2.39) and those who received the program via telehealth (*M* = 9.04, *SD* = 2.29), *F*(1, 41) = 6.71, *p* < 0.05, partial η^2^ = 0.14. A main effect of time also emerged, such that there was statistically significant difference in anxiety scores at baseline (*M* = 9.16, *SD* = 3.02), and at 15 weeks (*M* = 7.95, *SD* = 2.62), regardless of program delivery format, *F*(1, 41) = 7.44, *p* < 0.01, partial η^2^ = 0.15. The interaction did not emerge as significant. The groups (in-person vs. telehealth) did not differ significantly on any other measure.

## Discussion

This investigation of SWGP demonstrated high attendance, participant satisfaction, and likelihood to recommend the program. Findings suggest the program is acceptable in an outpatient cancer center whether delivered in person or via telehealth. Participants demonstrated a reduction in symptoms of anxiety and depression, increased perceived knowledge of nutrition, exercise, stress, sleep, emotional wellness, spiritual wellness, sexual wellness, and goal-setting, and improved quality of life from baseline to 15-week follow-up.

The American Society of Clinical Oncology, National Comprehensive Cancer Network (NCCN), American College of Surgeons, and the National Academy of Sciences offer several guidelines and recommendations for survivorship care [1–3, 7]. Cancer centers face challenges translating these guidelines into standardized, evidence-based clinical services that meet the diverse physical and psychological needs of cancer patients after treatment. Patients wish to learn how to manage late effects of treatments, reduce risk of recurrence, engage in healthy behaviors, and improve their quality of life [23, 24], yet many survivorship clinics and care plans are limited to surveillance for cancer spread, recurrence, and second cancers [25].

Research of psychological, behavioral, or lifestyle interventions for cancer survivors is heterogenous in design and outcome of interest (e.g., anxiety, fear of recurrence, and depressive symptoms; weight management, physical activity, and dietary behaviors; fatigue, insomnia, pain, and cognitive impairment; and return to work [26–28]). Existing multicomponent interventions often focus on diet, exercise, behavior modification, and stress management [17, 29], the majority of which have demonstrated acceptability, feasibility, and benefits to survivors.

SWGP presents a unique multidisciplinary model that integrates comprehensive survivorship care across multiple domains of physical, psychological, sexual, and spiritual wellness. Based on SWGP participants’ improvements in perceived knowledge across multiple topics, the breadth of needs the multidisciplinary care team addresses may not only be a defining aspect, but a strength of the program. SWGP is designed to reduce the burden placed on survivors to seek out separate providers and programs for each of many unmet needs, instead streamlining comprehensive survivorship care in a single service. This model addresses several care standards for cancer survivors, is relevant for cancer centers with and without centralized survivorship clinics [30] and is well-suited to supplement existing survivorship plans by targeting unmet health behavior, psychological, and overall wellness needs.

For many survivors, the post-treatment period involves feeling untethered from care providers, struggling with lingering effects of treatment, and declining interpersonal support [14, 31, 32]. SWGP was designed to support cancer patients during this critical transition from active treatment. In a multicenter longitudinal study of survivors who had received treatment for breast, prostate, colorectal, and gynecologic cancer, and non-Hodgkin's lymphoma, 30% reported more than five unmet supportive care needs immediately post-treatment, which did not improve six months later for 60% of respondents [33]. SWGP provides timely access by conducting enrollment on a rolling basis, offering patients flexibility to seek survivorship care at a time suitable to their needs and personal circumstances.

While SWGP addresses a multitude of survivorship needs, a central theme of the program is management of psychosocial distress post-active treatment, as emotional distress has been considered the “sixth vital sign” in cancer care [34]. The most frequently endorsed unmet supportive care needs in survivorship are fear of recurrence and other psychological concerns, including uncertainty about the future and worry that treatment results are beyond one’s control [33]. Indeed, the most common emotional reactions after cancer treatment are stress, anxiety, depression, and fear [35]. Psychological distress has also been associated with noncompliance with some NCCN-recommended cancer surveillance screening behaviors in long-term cancer survivors [36] and engagement in health-enhancing behaviors, such as exercise [37]. SWGP employs evidence-based cognitive-behavioral, mindfulness, and acceptance-based techniques to manage stress, uncertainty, fear of recurrence, and perceived loss of control, and shows promise in reducing symptoms of anxiety and depression among cancer survivors. These findings align with meta-analyses demonstrating that psycho-oncologic interventions are associated with significant effects on anxiety, depression, and quality of life among cancer survivors [38, 39].

Promotion of nutrition, physical activity, and sleep following cancer treatment is a critical component of survivorship care, and interventions for these are associated with improved quality of life [40–43]. SWGP utilizes several health behavior change strategies including use of SMART (specific, measurable, achievable, realistic, time-limited) goals, goal accountability, problem solving of barriers, and self-monitoring of behavior change efforts [44–47]. In this study, SWGP was associated with significant increases in physical, functional, and overall quality of life, and participants reported significant increases in perceived knowledge of several health behavior domains, including nutrition, exercise, sleep, sexual wellness, and goal-setting. While physiological measures of changes, such as those in weight or hours slept, were not obtained, participants reported improved perception of goal attainment.

Post-treatment challenges faced by survivors intensified with the COVID-19 pandemic, coinciding with the transition of SWGP to telehealth [19]. Participants who attended the program exclusively via telehealth reported elevated anxiety at baseline compared to those who attended in-person, and, while not statistically significant, the anxiety of telehealth participants did not lessen to the same degree as those who attended in-person. These findings align with unique circumstances related to the pandemic, including perceived heightened vulnerability to COVID-19, social isolation, and risk of disease complication, and concern about disruption of medical services [48–51]. No other outcomes emerged as significantly different between participants who received the intervention in-person versus telehealth, suggesting the program demonstrated preliminary efficacy and acceptability with either mode of delivery.

### Limitations

The current analyses were conducted retroactively on a convenient sample. We were not able to retroactively contact, and therefore, obtain consent and include all SWGP participants, and of those we successfully contacted and obtained consent from, some had not completed the 15-week follow-up questionnaire. These recruitment methods may have led to a biased sample. Additionally, lack of a control group limits the conclusions that can be drawn about the efficacy of SWGP compared to treatment as usual or time/attention control. Natural improvement in distress and quality of life over time may account for some of the improvement observed.

Also of concern is the limited diversity of the sample. Notably, our sample was predominantly female, white, and highly educated. While it is unclear why men were poorly represented in this sample, a large portion of SWGP patients were survivors of breast cancer (49%), which disproportionately affects women, potentially explaining this skew. Nonetheless, patients with breast and digestive system malignancies represent the two largest groups seen by our Cancer Center. Thus, engaging in targeted outreach to patients with male genital, urinary, and endocrine cancers is an important future direction. SWGP was conducted in San Francisco, California, where according to the United States census, only 5.7% and 15.9% of the population in this county identify as African American and Hispanic, respectively, percentages that closely mirror the demographic make-up of patients treated at UCSF Psycho-Oncology, which may be one contributing factor for low representation of these groups in the current sample [52]. It is unclear why highly educated individuals were drawn to SWGP. One potential explanation for this is the possibility that greater education correlates with greater awareness of and appreciation for the benefits of wholistic wellness during survivorship drawing individuals with greater education to a multidisciplinary wellness program like SWGP. Moreover, SWGP was offered in the afternoon on a weekday, limiting participation to patients who could be available during that time (e.g., individuals not working or with a flexible work schedule). Because most group-based psychosocial intervention studies report similar samples, representativeness and generalizability of results are pervasive concerns in this literature. This highlights the continued and critical need to actively encourage individuals of underrepresented groups to participate in clinical programs, removing barriers whenever possible. Offering programs by telehealth may be one way to reduce barriers and potentially increasing engagement among underrepresented samples.

Lastly, we did not collect qualitative data on perceived goal attainment or data on referrals to/from SWGP versus individualized care clinics (e.g., nutrition, sleep). This lack of data limits our ability to understand why only 47% of the sample reported meeting their goals and if the multidisciplinary nature of SWGP streamlined referral to individual care clinics while reducing patient burden as intended. Patients may have reported low goal attainment for several reasons: 1) patients were asked about goals, but were not required to formally track completion, 2) with only one session per topic, patients may have been challenged to reach a specific goal in such limited time. It remains unknown if and which participants sought additional care and for what concern. Importantly, it is possible patients in the current sample were also receiving individualized care in one or more individual care clinics (e.g., nutrition, sleep) while participating in SWGP, thereby, potentially conflating the results. Findings from the current analyses should, therefore, be interpreted with this consideration in mind.

### Conclusion

This study demonstrates that SWGP promotes wellbeing, reduces self-reported mental health symptoms, increases perceived knowledge of health and wellness, and is acceptable whether delivered in-person or telehealth. SWGP is reimbursable by insurance, suggesting the model may be financially sustainable. SWGP may offer a replicable model for cancer centers to meet national cancer care standards and guidelines for cancer survivors at a critical transition in care.

## Data Availability

The datasets generated during the current study and analyzed for the article are available from the corresponding author on reasonable request.
